# Cyclin C Regulated Oxidative Stress Responsive Transcriptome in *Mus musculus* Embryonic Fibroblasts

**DOI:** 10.1534/g3.119.400077

**Published:** 2019-04-29

**Authors:** David C. Stieg, Kai-Ti Chang, Katrina F. Cooper, Randy Strich

**Affiliations:** Department of Molecular Biology, Graduate School of Biological Sciences, Rowan University, Stratford, NJ 08084

**Keywords:** Cyclin C, Oxidative Stress, RNA-seq, Cdk8, Mediator

## Abstract

The transcriptional changes that occur in response to oxidative stress help direct the decision to maintain cell viability or enter a cell death pathway. Cyclin C-Cdk8 is a conserved kinase that associates with the RNA polymerase II Mediator complex that stimulates or represses transcription depending on the locus. In response to oxidative stress, cyclin C, but not Cdk8, displays partial translocation into the cytoplasm. These findings open the possibility that cyclin C relocalization is a regulatory mechanism governing oxidative stress-induced transcriptional changes. In the present study, the cyclin C-dependent transcriptome was determined and compared to transcriptional changes occurring in oxidatively stressed *Mus musculus* embryonic fibroblasts. We observed a similar number (∼2000) of genes up or downregulated in oxidatively stressed cells. Induced genes include cellular repair/survival factors while repressed loci were generally involved in proliferation or differentiation. Depleting cyclin C in unstressed cells produced an approximately equal number of genes (∼2400) that were repressed by, or whose transcription required, cyclin C. Consistent with the possibility that cyclin C nuclear release contributes to transcriptional remodeling in response to oxidative stress, we found that 37% cyclin C-dependent genes were downregulated following stress. Moreover, 20% of cyclin C- repressed genes were induced in response to stress. These findings are consistent with a model that cyclin C relocalization to the cytoplasm, and corresponding inactivation of Cdk8, represents a regulatory mechanism to repress and stimulate transcription of stress-responsive genes.

Reactive oxygen is a common cytotoxic compound that targets many cellular compartments and macromolecules (Avery 2011). In response to oxidative stress, the cell must decide whether to undergo a transient growth arrest to repair the damage or enter a cell death pathway (Giannattasio, Guaragnella, Arbini, & Moro 2013; Han *et al.*, 2008). To accomplish either cell fate, the transcriptional program must be altered to repress growth-promoting genes, while inducing loci encoding damage control proteins such as chaperones or detoxifying enzymes (Ma 2010). In either case, the activity of transcriptional inducers and repressors of these genes must themselves be modulated. Cyclin C, along with its cognate cyclin-dependent kinase 8 (Cdk8), are components of the highly conserved Cdk8-Kinase Module (CKM). The CKM, also containing Med12 and Med13, interacts transiently with the mediator complex (Bourbon 2008) and plays both a positive and negative role in transcription (Nemet, Jelicic, Rubelj, & Sopta 2014). For example, the CKM activates the β-catenin signaling pathway in colorectal cancer cells (Firestein *et al.*, 2008) and functions as a coactivator with p53 to induce the Cdk inhibitor p21 (Donner, Szostek, Hoover, & Espinosa 2007). Conversely, the CKM represses transcription by modifying cyclin H of TFIIH (Akoulitchev, Chuikov, & Reinberg 2000) and inducing the degradation of the intracellular domain (ICD) involved in Notch signaling (Fryer, White, & Jones 2004). In support of the importance of the CKM in ICD degradation, cyclin C has recently been identified as a tumor suppressor due to this activity (Li *et al.*, 2014). In mammalian cells, paralogs of Med12 (Med12L) and Med13 (Med13L) also associate with cyclin C and Cdk8 but no specific function has been described for these different CKM compositions (Allen & Taatjes 2015). In the budding yeast *S. cerevisiae*, the CKM components play very similar roles primarily as transcriptional repressors (van de Peppel *et al.*, 2005). However, this may not be the case during development in higher organisms (Li *et al.*, 2014; Loncle *et al.*, 2007; Westerling, Kuuluvainen, & Makela 2007).

Although its role is more varied, the budding yeast CKM was initially identified as a repressor of stress responsive (Cooper, Mallory, Smith, & Strich 1997; Holstege *et al.*, 1998) and meiotic (Cooper *et al.*, 1997; Surosky, Strich, & Esposito 1994) genes. To relieve this repression in response to oxidative stress, cyclin C translocates from the nucleus to the cytoplasm where it is destroyed (Cooper *et al.*, 2012). Preventing cyclin C relocalization and destruction decreases, but does not eliminate, gene induction (Cooper *et al.*, 1997; Cooper *et al.*, 2012). Prior to its proteolysis, the yeast cyclin C relocalizes to the mitochondria, where it is necessary and sufficient for oxidative stress-induced fragmentation of this organelle (Cooper, Khakhina, Kim, & Strich 2014; Cooper *et al.*, 2012) and programmed cell death (PCD) (Cooper *et al.*, 2014). This cytoplasmic role for cyclin C in both mitochondrial fission and PCD is conserved in mammalian cells (Ganesan *et al.*, 2018; Wang, Yan, Cooper, & Strich 2015). However, rather than all of cyclin C translocating to the cytoplasm as observed in stressed yeast cells, only about 20% of total cyclin C is released following oxidative stress in mammalian cells (Wang *et al.*, 2015). Cyclin C nuclear release in yeast requires destruction of CKM component Med13 via the SCF ubiquitin ligase mediated by the F-box protein Grr1 (Stieg *et al.*, 2018). Interestingly, the normal turnover of mammalian Med13 is dependent on the F-box protein Fbw7 (Davis *et al.*, 2013), an ortholog of Grr1. Therefore, Med13 destruction in mammalian systems may be more selective allowing the precise removal of cyclin C, and thereby inhibiting Cdk8 activity, from specific promoters in response to stressors such as reactive oxygen.

In this study, we combined a bioinformatics approach with *Ccnc^+/+^* and *Ccnc^−/−^* immortalized mouse embryonic fibroblasts (MEFs) to identify genes repressed or induced in response to oxidative stress exposure, and the role of cyclin C in the transcriptional regulation of those genes. These studies revealed that 20-30% of stress-repressed or induced genes is also controlled by cyclin C. In addition, the results point to a potential role for cyclin C nuclear to cytoplasmic shuttling in mediating the correct cellular response to oxidative stress exposure.

## Materials and Methods

### Cell culture

*Ccnc*^+/+^ (WT) and *Ccnc^−/−^* (Wang *et al.*, 2015) immortalized MEF cells were cultured in Dulbecco’s Modified Eagle Medium (DMEM) supplemented with 10% fetal bovine serum (FBS) and 1% penicillin/streptomycin at 37° and 5% CO_2_. Replicate WT and *Ccnc^−/−^* MEF cultures were grown to 80% confluence and treated as follows. Oxidative stressed cells were exposed to 0.4 mM H_2_O_2_ in DMEM without FBS or antibiotics for 4 h. Control unstressed WT MEFs were treated with DMSO in DMEM without FBS and antibiotics for 4 h. Following treatments, cells were harvested using trypsin and RNA was extracted as described below. All experiments were conducted in triplicate for each condition.

### Total RNA extraction and RNA-seq

Total RNA was prepared from the cell samples using mechanical disruption and the QIAGEN RNeasy kit. RNA quality and quantity were assessed using a Nanodrop UV Spectrophotometer (ThermoFisher). Total RNA from each sample underwent further QC analysis including NanoDrop2000, Qubit analysis and Agilent RNA ScreenTape System/TapeStation (GENEWIZ). The RNA integrity numbers (RIN) for all samples ranged from 8.5-10. The RNA was enriched for mRNA using polyA+ selection and a barcoded cDNA library was prepared for each sample. The barcoded cDNA libraries were sequenced using an Illumina HISEQ 2500 instrument to produce ~25 million paired-end reads for each sample. The raw data from the sequencing were converted to FASTQ files for further analysis.

### Mapping RNA-seq reads

The quality of each FASTQ file was assessed using FastQC then uploaded into the Galaxy environment (Afgan *et al.*, 2018) for all subsequent analyses. The forward and reverse FASTQ files for each sample were paired and grouped into the following collections: WT, WT Stress, *Ccnc^−/−^*. The paired-end reads were mapped to the Ensembl *Mus musculus* (GRCm38) cDNA FASTA file downloaded from UCSC (Casper *et al.*, 2018). Mapping was performed with Bowtie2 (Langmead & Salzberg 2012) using default settings. The Bowtie2 mapping statistics for the indicated samples are listed in Table S1. To generate count tables from the reads uniquely mapped to annotated genes, featureCounts was performed on each sample using the GRCm38 annotation gtf file. The featureCounts data were filtered for protein coding genes and aggregated for gene level analysis.

### DGE analysis

The gene level data produced by featureCounts were used to determine differential gene expression (DGE) between samples. DGE analysis identifies genes that display statistically significant changes in transcript abundance between experimental conditions. DGE analyses were completed using DESeq2 (Anders & Huber 2010) on the following group comparisons: WT:WT Stress; WT:*Ccnc^−/−^*. DESeq2 uses a negative binomial generalized linear model to test for significance (p-value) using the Wald test. Automatic filtering is incorporated into DESeq2 to eliminate low abundance genes from DGE analysis. The Benjamini & Hochberg p-value correction was used to produce adjusted p-values, or false discovery rates (FDRs), by which the DGE analysis results were filtered (FDR < 0.05). The filtered data from DESeq2 were used to create datasets of up and down DGE in each of the experimental conditions. The datasets were compared to create sets of overlapping induced and repressed genes in the differing experimental conditions. Data from the DGE analyses are listed in Table 1.

**Table 1:** Number of genes differentially expressed in *Ccnc^−/−^* and WT Stress MEFs, in comparison to WT MEFs. Also included is the number of genes unique to each condition in the corresponding comparison

Comparsion	DGE (# of genes)	Unique to WT Control (# of genes)	Unique to WT Stress (# of genes)	Unique to CCNC KO Control (# of genes)
WT: WT_Stress_	**3,841**	**45**	**444**	
WT: *Ccnc*^−/−^	**4,835**	**160**		**298**
Overlap Between Previous Two Comparisons	**1,375**			

DGE: differentially expressed genes (FDR-adjusted P-value < 0.05).

Unique: absence of gene expression in the opposing condition.

### GO analysis

Gene ontology (GO) analysis (Ashburner *et al.*, 2000; The Gene Ontology Consortium 2017) on groups of genes identified from RNA-seq analysis was completed using the Functional Annotation Tool in the Database for Annotation, Visualization and Integrated Discovery (DAVID) (Huang da, Sherman, & Lempicki, 2009a, 2009b). The data collected from the Functional Annotation search included gene groups identified in a specific biological process (GOTERM BP DIRECT), molecular function (GOTERM MF DIRECT), cellular compartment (GOTERM CC DIRECT), Uniprot keywords (UP KEYWORDS), and KEGG pathway term (KEGG PATHWAY). Functional Annotation Clustering uses an algorithm to reduce redundant term association and provides an enhanced biological interpretation of specific gene groups being analyzed.

### RT-qPCR validation of results

Total RNA was extracted from triplicate MEF cultures described in the text then converted to cDNA using the ThermoFisher Maxima cDNA Synthesis kit. On-column DNase treatment was performed to eliminate contaminating DNA. The cDNA from each sample was subjected to qPCR amplification using ThermoFisher PowerSYBR Green PCR Master Mix and a StepOne Real Time PCR System. Primer sequences are provided in Table S9. These assays were conducted with three independent preparations assayed in duplicate. *Gapdh* was used as the internal standard for comparative (ΔΔC_T_) quantitation (Livak & Schmittgen 2001). The analysis of unstressed WT MEFs was completed as a control in the experiments.

### Data Availability

The raw data, processed data and metadata for this project were submitted to GEO (Barrett *et al.*, 2013; Edgar, Domrachev, & Lash 2002). The GEO series accession is GSE126450. Supplemental material available at FigShare: https://doi.org/10.25387/g3.8048756.

## Results

### Oxidative stress response in the WT culture

**Figure 1:**
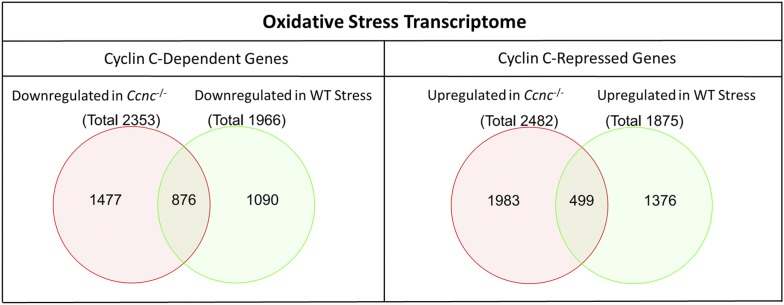
Venn Diagrams of overlapping genes between the two comparisons: WT: *Ccnc*^−/−^; WT: WT_Stress_. The diagram on the left displays the overlapping genes that are downregulated in both conditions (Cyclin C-Dependent; 876 genes). The diagram on the right displays the overlapping genes that are upregulated in both conditions (Cyclin C-Repressed; 499 genes).

The RNA-seq results were analyzed as described in Materials and methods to identify transcriptional changes occurring in WT cells in response to H_2_O_2_ treatment by comparing WT and WT Stress. The featureCounts data from each group were analyzed using the DESeq2 algorithm. Filtering the DESeq2 results for significance (FDR < 0.05) produced a final dataset including 3,841 genes that were differentially expressed between WT Control and WT Stress cells. The filtered WT:WT_stress_ dataset identified a nearly identical number of genes exhibiting downregulated (1,966) or upregulated (1,875) mRNA levels (Figure 1). The 50 genes exhibiting the most reduced or increased values are provided in Table S2.

The datasets of repressed and induced genes in WT stressed cells were further analyzed using the Functional Annotation Tool in DAVID (Huang da *et al.*, 2009a, 2009b). To focus on specific genes, the enriched clusters produced by DAVID Functional Annotation Clustering were analyzed. The results of the analysis with the highest enrichment scores for oxidative stress-repressed and induced groups are presented in Tables S3 and S4, respectively. The clustering analysis of the oxidative stress-repressed genes included enriched clusters for processes or functions involved in cell adhesion, kinase activity/phosphorylation, cell cycle/cell division and oxidoreductase activity. The clustering analysis of the oxidative stress-induced genes included enriched clusters for processes or functions involved in DNA damage response, DNA repair, ubiquitin conjugation, autophagy/autophagosome and protein transport. In agreement with previous oxidative stress transcriptome studies (Han *et al.*, 2008), we observed a downregulation in genes involved in cell cycle, cell adhesion and signal transduction, and an upregulation in genes involved in DNA damage, ubiquitin-mediated proteolysis and autophagy. Therefore, in response to oxidative stress, there is a dramatic shift from the transcription of genes required for growth and differentiation toward expression of genes involved in cellular repair, survival and cell death pathways.

### Cyclin C-dependent transcriptome

To understand the role of cyclin C-Cdk8 in transcription, RNA-seq was performed on mRNA prepared from unstressed *Ccnc^−/−^* MEFs then compared to the wild-type transcriptome just described. The featureCounts data for each group were subjected to DGE analysis using DESeq2. In contrast to previous studies (Li *et al.*, 2014), our results indicate a larger disruption of transcriptional regulation resulting from cyclin C deletion. The filtered results (FDR < 0.05) included a total of 4,835 genes differentially expressed between WT and *Ccnc^−/−^* MEFs with a similar partition of downregulated (2,353) or upregulated (2,482) genes (Figure 1). This relative even distribution between cyclin C-dependent and repressed loci was also observed in unstressed cells inhibited for Cdk8 activity (Galbraith *et al.*, 2017). The 50 genes exhibiting the largest change in mRNA levels either up or down in the absence of cyclin C are shown in Table S5. The genes downregulated in *Ccnc^−/−^* MEFs formally indicate a positive role for this factor and will be referred to as the cyclin C-dependent gene set. Similarly, the genes whose levels increase in *Ccnc^−/−^* MEFs will be considered the cyclin C-repressed gene set.

The datasets of cyclin C-dependent and repressed genes were analyzed using DAVID as described above. The results obtained from Functional Annotation Clustering were used to identify enriched groups in both gene sets. The results of the clustering analysis with the highest enrichment for cyclin C-dependent and repressed genes are shown in Tables S6 and S7, respectively. Enriched clusters for cyclin C-dependent genes include mitochondrial proteins, oxidoreductase activity, cell adhesion and cell cycle/cell division. This result suggests an important role for cyclin C-dependent genes in cellular energy production, movement, cell proliferation and propagation. Clustering analysis for cyclin C-repressed genes included protein degradation pathways, protein transport and cell proliferation pathways. This suggests cyclin C-repressed genes are involved in protein degradation pathways and negative regulation of proliferation and differentiation. The data demonstrate that cyclin C regulates the transcription of select genes involved in energetics, protein metabolism and cell cycle control.

### Analysis of overlapping gene sets

The next step of the analysis was to determine whether an overlap existed between the oxidative stress and cyclin C regulated gene data sets. First, we examined the data overlap for cyclin C-dependent genes with those whose mRNA levels were reduced in stressed cells. This comparison produced an overlap of 876 genes that fell into this category (Figure 1). This represented 37% of the total number of cyclin C-dependent genes identified. On the other hand, 499 cyclin C-repressed genes were upregulated following oxidative stress, representing 20% of total cyclin C-repressed genes prior to stress. These results indicate that the cyclin C transcriptome contains a large amount of genes responsive to oxidative stress.

The overlapping datasets just described were further clustered for GO terms using the Functional Annotation Tool in DAVID. The highest enriched groups for cyclin C- dependent/oxidative stress repressed data set are shown in Table 2. The clustering analysis revealed enriched pathways including oxidoreductase activity, lipid metabolism, cell adhesion, cell proliferation pathways and multicellular organism development. The genes in the enriched groups can be found in Table S8, with the DGE data for each gene. The clustering analysis for cyclin C-repressed/oxidative stress induced genes identified groups enriched for protein transport, cellular response to DNA damage stimulus and DNA repair, lysosomal proteins, ubiquitin conjugation and unfolded protein response pathways (Table 3). The individual gene results for this data set are found in Table S8. These results indicate that cyclin C-dependent genes whose expression is reduced following oxidative stress, are involved cellular energy production, movement and cell proliferation. These results also indicate that cyclin C-repressed genes whose expression is increased in response to stress, are involved in protein transport, stress response gene activation and protein degradation. Taken together, these results suggest that cyclin C inactivation is an important component in the complex regulatory network controlling the oxidative stress transcriptome.

**Table 2:** Cyclin C-Dependent Genes. GO-Functional Annotation Clusters for cyclin C-dependent genes. The following clusters (1-5) resulting from DAVID-GO Functional Annotation Clustering, represent the overlapping genes downregulated in both WT Stress and *Ccnc^−/−^* MEFs (876 genes)

Cyclin C-Dependent Genes					
Cluster	ES	Category	Associated Term	p-value	Genes Involved (#)
**1**	**6.16**	UP KEYWORDS	Oxidoreductase	1.08E-7	54
		GOTERM BP DIRECT	Oxidation-reduction process	7.10E-7	58
		GOTERM MF DIRECT	Oxidoreductase activity	1.69E-6	53
**2**	**5.94**	UP KEYWORDS	Lipid metabolism	2.65E-7	40
		GOTERM BP DIRECT	Lipid metabolic process	4.72E-7	45
		UP KEYWORDS	Lipid biosynthesis	1.18E-5	20
**3**	**5.17**	GOTERM MF DIRECT	Cadherin binding involved in cell-cell adhesion	1.44E-6	32
		GOTERM CC DIRECT	Cell-cell adherens junction	3.70E-6	33
		GOTERM BP DIRECT	Cell-cell adhesion	5.89E-5	22
**4**	**3.91**	KEGG PATHWAY	Rap 1 signaling pathway	7.73E-7	29
		KEGG PATHWAY	P13K-Akt 1 signaling pathway	2.40E-4	33
		KEGG PATHWAY	Ras signaling pathway	1.18E-2	20
**5**	**3.67**	UP KEYWORDS	Differentiation	9.10E-6	49
		UP KEYWORDS	Developmental protein	1.90E-4	61
		GOTERM BP DIRECT	Multicellular organism development	5.72E-3	62

ES = Enrichment score produced by Functional Annotation Clustering in DAVID.

Category Terms Defined: UP Keywords = Uniprot Keywords; GOTERM BP DIRECT = GO Term for Direct Involvement in Biological Process; GOTERM MF DIRECT = GO Term for Direct Involvement in Molecular Function; GOTERM CC DIRECT = GO Term for Direct Localization to Cellular Compartment; KEGG PATHWAY = KEGG Pathway.

**Table 3:** Cyclin C-Repressed Genes. GO-Functional Annotation Clusters for cyclin C-repressed genes. The following clusters (1-5) resulting from DAVID-GO Functional Annotation Clustering, represent the overlapping genes upregulated in both WT Stress and *Ccnc^−/−^* MEFs (499 genes)

Cyclin C-Repressed Genes					
Cluster	ES	Category	Associated Term	p-value	Genes Involved (#)
**1**	**3.69**	UP KEYWORDS	Protein transport	4.46E-6	32
		GOTERM BP DIRECT	Protein transport	6.48E-6	34
		UP KEYWORDS	Transport	6.02E-3	59
		GOTERM BP DIRECT	Transport	1.03E-2	60
**2**	**3.39**	UP KEYWORDS	DNA damage	5.54E-5	21
		GOTERM BP DIRECT	Cellular response to DNA damage stimulus	8.19E-5	25
		GOTERM BP DIRECT	DNA repair	1.80E-3	18
**3**	**3.14**	GOTERM CC DIRECT	Lysosomal membrane	3.16E-5	18
		UP KEYWORDS	Lysosome	1.22E-3	15
		GOTERM CC DIRECT	Lysosome	1.01E-2	16
**4**	**3.13**	GOTERM BP DIRECT	Protein ubiquitination	7.44E-6	25
		UP KEYWORDS	Ubl conjugation pathway	6.24E-5	31
		GOTERM MF DIRECT	Ubiquitin-protein transferase activity	2.68E-2	15
**5**	**2.10**	UP KEYWORDS	Unfolded protein response	1.09E-5	8
		GOTERM BP DIRECT	Response to unfolded protein	2.00E-4	8

ES = Enrichment score produced by Functional Annotation Clustering in DAVID.

Category Terms Defined: UP Keywords = Uniprot Keywords; GOTERM MF DIRECT = GO Term for Direct Involvement in Molecular Function; GOTERM BP DIRECT = GO Term for Direct Involvement in Biological Process; GOTERM CC DIRECT = GO Term for Direct Localization to Cellular Compartment.

### RT-qPCR validation

**Figure 2:**
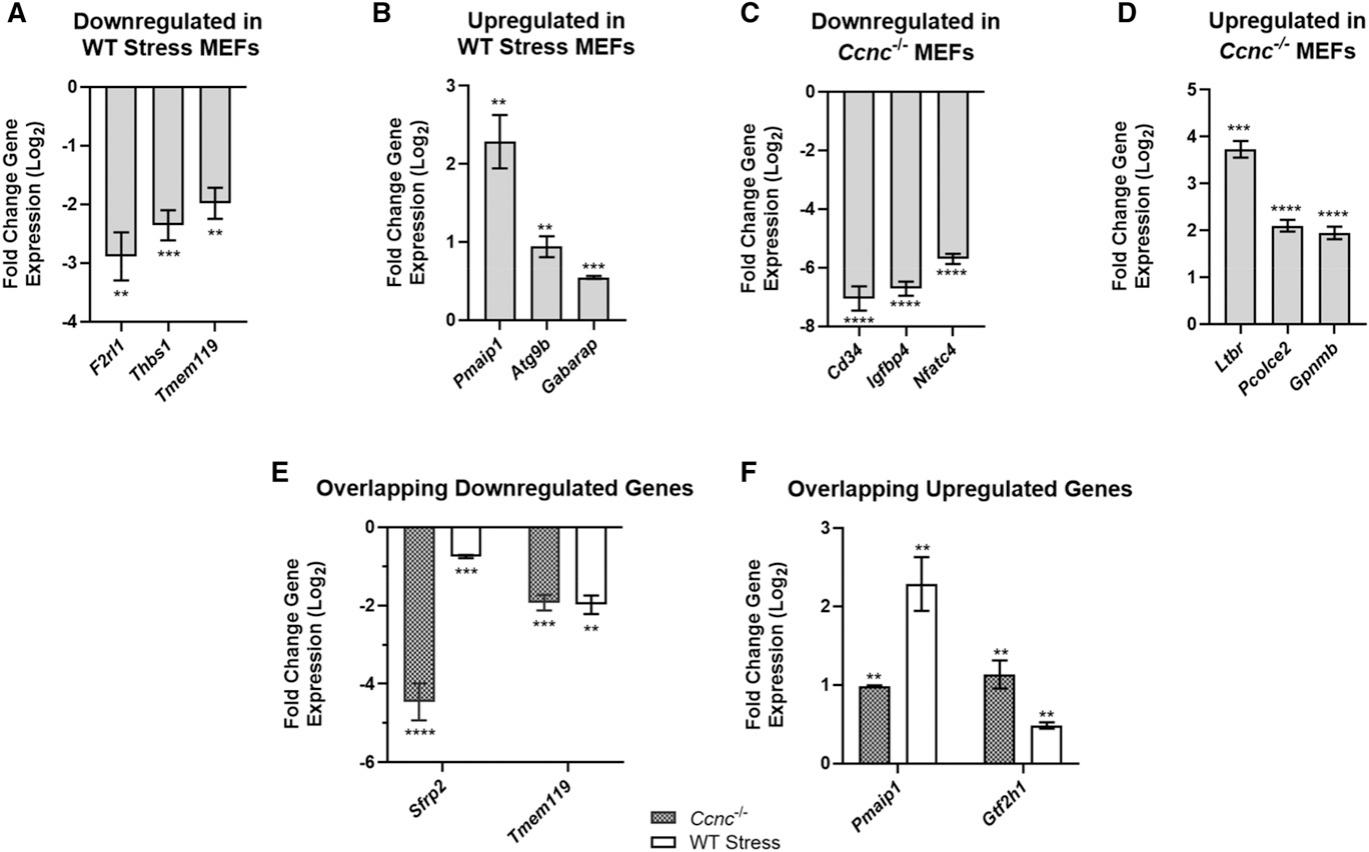
RT-qPCR Validation of RNA-seq Data. RT-qPCR analysis results for relative fold change expression (log_2_) values using unstressed WT MEFs as a control for both (A) downregulated genes in WT Stress MEFs (*F2rl1*, *Thbs1*, *Tmem119)*, and (B) upregulated genes WT Stress MEFs (*Pmaip1*, *Atg9b*, *Gabarap)* and (C) downregulated genes in *Ccnc*^−/−^ MEFs (*Cd34*, *Igfbp4*, *Nfatc4*), and (D) upregulated genes in *Ccnc*^−/−^ MEFs (*Gpnmb*, *Pcolce2*, *Ltbr*). RT-qPCR analysis for relative fold change expression (log_2_) values for both (E) overlapping downregulated genes (*Sfrp2*, *Tmem119*), and (F) overlapping upregulated genes (*Pmaip1*, *Gtf2h1*) in WT Stress and *Ccnc*^−/−^ MEFs. Error bars represent SEM (*p value < 0.05, **p-value < 0.01, ***p-value < 0.001, ****p-value < 0.0001).

To validate the RNA-seq results, the levels of selected mRNAs identified as significantly changed following bioinformatic analyses were interrogated using RT-qPCR. Total RNA preparations from each sample were used to synthesize cDNA for qPCR analysis. Quantitation of each mRNA was achieved using glyceraldehyde 3-phosphate dehydrogenase *Gapdh* as an internal reference standard. The delta-delta-CT (∆∆CT) method was used to determine transcript levels of each gene (Livak & Schmittgen 2001). The results of the qPCR analyses are shown in Figure 2. In WT stressed MEFs, qPCR analysis verified the RNAseq results for three downregulated genes (*F2rl1*, *Thbs1*, *Tmem119*) and three upregulated genes (*Pmaip1*, *Atg9b*, *Gabarap*). In *Ccnc^−/−^* MEFs, qPCR analysis verified three downregulated genes (*Cd34*, *Igfbp4*, *Nfatc4*), and three upregulated genes *(Ltbr*, *Pcolce2*, *Gpnmb*). In addition, qPCR analysis was used to verify overlapping downregulated genes (*Sfrp2*, *Tmem119*), and overlapping upregulated genes (*Pmaip1*, *Gtf2h1*). The results obtained from qPCR are in agreement with our RNA-seq data identifying differentially expressed genes following exposure to oxidative stress or change in cyclin C status.

## Discussion

Comprehensive expression analysis of the CKM in yeast indicated that cyclin C and Cdk8 performed primarily a repressive function on stress responsive genes, although positive roles for this kinase in transcription were noted (van de Peppel *et al.*, 2005). In this study, RNA-seq and bioinformatics were used to identify genes differentially expressed in response to H_2_O_2_ treatment and cyclin C status. Analysis of these data sets revealed a significant overlap between enriched GO clusters of cyclin C-dependent genes (*e.g.*, energy production, proliferation and development) that were also observed in oxidative stress-repressed genes. In contrast, cyclin C represses genes involved in protein transport, stress response gene activation and protein degradation, which are induced in response to H_2_O_2_ treatment. In stressed cells, these loci are induced to evoke repair or cell death pathways. These data indicate that cyclin C both represses and induces genes regulated by the oxidative stress response. In addition, our results suggest a model that cyclin C-Cdk8 inactivation plays an important role in mediating the transcriptional response to oxidative stress.

The results presented here indicate that cyclin C-Cdk8 plays a relatively equal role as a repressor or activator. This is consistent with previous reports examining MEFs deleted for *Ccnc* (Li *et al.*, 2014) or treated with a Cdk8 inhibitor (Galbraith *et al.*, 2017). An important caveat to this conclusion is that it has yet to be determined the percentage of these loci that are directly controlled by cyclin C-Cdk8. As transcription factors are included in the genes regulated by cyclin C-Cdk8 (*e.g.*, *Foxg1*, *Hoxb4*), there may be indirect effects of deleting *Ccnc*. The enriched clusters of cyclin C-dependent genes (Table S8) include plakoglobin (*Jup)*, which is involved in vascular endothelial-cadherin adhesion (Nottebaum *et al.*, 2008), and fibroblast growth factor 10 (*Fgf10*) involved in development and differentiation (Ohuchi 2000). In contrast, enriched clusters from cyclin C-repressed genes (Table S8) include *Jmy*, which encodes a protein involved in the p53-mediated response to cellular stresses and the activation of the *Bax* pro-apoptotic gene (Shikama *et al.*, 1999). Interestingly, our laboratory discovered that when released from the nucleus, cyclin C interacts with both the fission machinery (Ganesan *et al.*, 2018) and the pro-apoptotic factor Bax (J. Jezek, R. Strich, unpublished). Therefore, these results suggest that cyclin C removal from the nucleus stimulates Bax activity in two ways, transcriptionally and through direct interaction. Another gene included in the enriched clusters for cyclin C-repressed genes is *Znrf3*, which encodes an E3 ubiquitin ligase targeting Wnt signaling receptors, and as a result diminishes Wnt signaling activity (Hao *et al.*, 2012). Overexpression of Cdk8 is associated with enhanced Wnt/β-catenin signaling in colon cancer (Firestein *et al.*, 2008). These findings suggest a possible mechanism consistent with this observation that elevated Cdk8 activity reduces Znrf3 levels, allowing enhanced Wnt signaling. Another enriched cyclin C-repressed gene is *Gtf2h1*, which encodes a component of the TFIIH core complex involved in RNA transcription from RNA polymerase II (RNAP II) and transcription-coupled nucleotide excision repair of damaged DNA (Kershnar, Wu, & Chiang 1998). Since the CKM is known to repress transcription through TFIIH modification (Akoulitchev *et al.*, 2000), these results suggest an additional layer of transcriptional control by cyclin C in unstressed cells. Therefore, cyclin C removal from the nucleus may play a role in derepression of both RNAP II transcription and induction of genes required for increased transcriptional activity.

**Figure 3:**
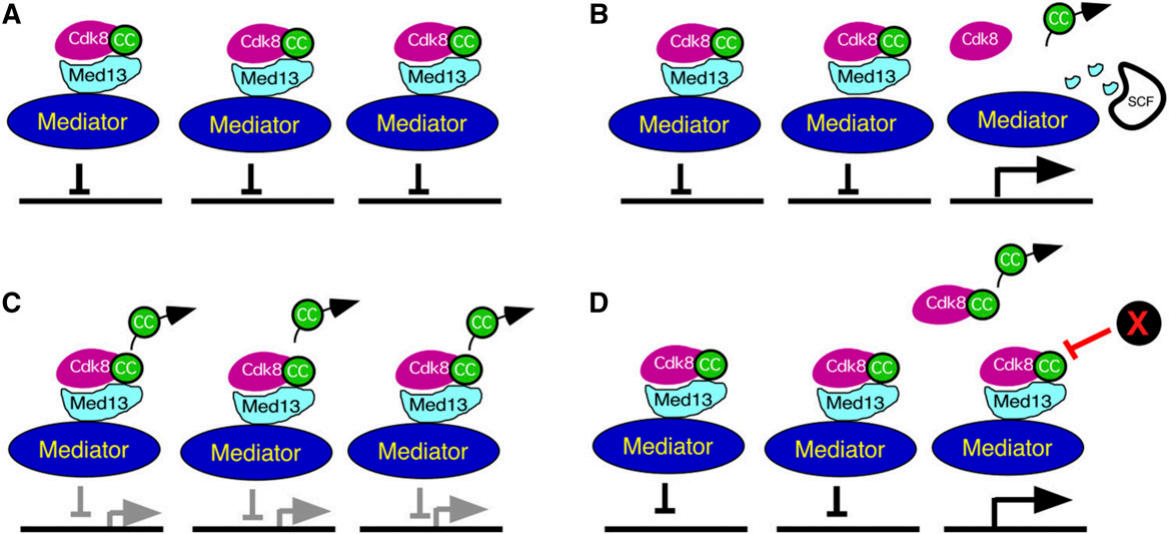
Cyclin C promoter release models. (A) A repressor model in unstressed cells with the CKM associated with the mediator controlling three different genes. Three potential models for selecting cyclin C for nuclear release are presented. (B) Model 1: Cyclin C release occurs primarily from a specific subset of promoters. This model predicts that targeted destruction of Med13 via recognition by the F-box protein Grr1 in the SCF (Skp-cullin-F-box) complex permits cyclin C nuclear release. Loss of cyclin C occupancy inactivates Cdk8, thus permitting normal transcription induction. (C) Model 2: Cyclin C is released from all promoters and this release may only be partially responsible for stress gene induction. Gray arrows and bars indicate partial derepression of cyclin C-Cdk8 repressed loci. (D) Model 3: Cyclin C is derived from a pool of the CKM not associated with the mediator. This model predicts that cyclin C-Cdk8 activity is inhibited by an unknown mechanism (“X”) independent of cyclin C promoter occupancy.

Relocalization to the cytoplasm is a common method to inactivate a transcription factor (Zanella, Dos Santos, & Link 2013). Consistent with the general repressor role for cyclin C-Cdk8 in yeast, we found that the complete cyclin C degradation in response to oxidative stress was required for normal stress gene induction (Cooper *et al.*, 2012). However, cyclin C-Cdk8 transcriptional control appears more complex in mammals. For example, we demonstrated that only ∼15–20% of cyclin C is released from the nucleus in response to oxidative stress (Wang *et al.*, 2015). This finding is consistent with our results that most genes controlled by cyclin C are not altered in response to oxidative stress. However, it is not understood how this pool of cyclin C is selected for nuclear release in response to stress. Three possible mechanisms seem most likely to explain this observation. In the simplest scenario, cyclin C-Cdk8 is tethered to the mediator by Med13 to mediate transcriptional repression (Figure 3A). In one model, cyclin C is released from specific loci through targeted destruction of Med13 (Figure 3B). We previously found that stress-activated signal transduction pathways directly trigger Med13 proteolysis via the ubiquitin-proteasome system (Stieg *et al.*, 2018). This specific removal of cyclin C would then inactivate Cdk8 to allow derepression of stress-induced genes. Similarly, growth-promoting genes dependent on cyclin C-Cdk8 would also be suppressed. Another possibility is that cyclin C is randomly selected from all promoters (Figure 3C). For repressed genes, partial removal of cyclin C would be predicted to result in partial derepression. However, we found that the majority of cyclin C repressed genes are not affected by oxidative stress suggesting that this possibility is less likely. Finally, it has been suggested that a small percentage of the CKM is not bound to the mediator (Knuesel, Meyer, Donner, Espinosa, & Taatjes 2009). Therefore, this potential free fraction of the CKM may be the source of cyclin C for cytoplasmic translocation (Figure 3D). In this scenario, another mechanism would be needed to inactive Cdk8 at the repressed promoters independent of cyclin C occupancy (“X” in Figure 3D). Further studies are required to confirm the occupancy of cyclin C at particular promoters before and after oxidative stress to determine which of these models is correct. Regardless of the mechanism involved, the secondary role of cyclin C in stimulating both mitochondrial fragmentation and apoptosis provides crosstalk between the nucleus and the mitochondria. Such a system would allow the cell to coordinate changes in the transcription program with the cell death pathway at the mitochondria.
